# Duloxetine versus ‘active’ placebo, placebo or no intervention for major depressive disorder; a protocol for a systematic review of randomised clinical trials with meta-analysis and trial sequential analysis

**DOI:** 10.1186/s13643-021-01722-5

**Published:** 2021-06-09

**Authors:** Faiza Siddiqui, Marija Barbateskovic, Sophie Juul, Kiran Kumar Katakam, Klaus Munkholm, Christian Gluud, Janus Christian Jakobsen

**Affiliations:** 1 Copenhagen Trial Unit, Centre for Clinical Intervention Research, The Capital Region, Rigshospitalet, Copenhagen University Hospital, Copenhagen, Denmark; 2 Stolpegaard Psychotherapy Centre, Mental Health Services in the Capital Region of Denmark, Gentofte, Denmark; 3 Department of Psychology, University of Copenhagen, Copenhagen, Denmark; 4 Centre for Evidence-Based Medicine Odense (CEBMO) and Cochrane Denmark, Department of Clinical Research, University of Southern Denmark, Odense, Denmark; 5 Open Patient Data Exploratory Network (OPEN), Odense University Hospital, Odense, Denmark; 6 Department of Regional Health Research, The Faculty of Heath Sciences, University of Southern Denmark, Odense, Denmark

**Keywords:** Adverse effects, Anti-depressants, Duloxetine, Meta-analysis

## Abstract

**Background:**

Major depression significantly impairs quality of life, increases the risk of suicide, and poses tremendous economic burden on individuals and societies. Duloxetine, a serotonin norepinephrine reuptake inhibitor, is a widely prescribed antidepressant. The effects of duloxetine have, however, not been sufficiently assessed in earlier systematic reviews and meta-analyses.

**Methods/design:**

A systematic review will be performed including randomised clinical trials comparing duloxetine with ‘active’ placebo, placebo or no intervention for adults with major depressive disorder. Bias domains will be assessed, an eight-step procedure will be used to assess if the thresholds for clinical significance are crossed. We will conduct meta-analyses. Trial sequential analysis will be conducted to control random errors, and the certainty of the evidence will be assessed using GRADE. To identify relevant trials, we will search Cochrane Central Register of Controlled Trials, Medical Literature Analysis and Retrieval System Online, Excerpta Medica database, PsycINFO, Science Citation Index Expanded, Social Sciences Citation Index, Conference Proceedings Citation Index—Science and Conference Proceedings Citation Index—Social Science & Humanities. We will also search Chinese databases and Google Scholar. We will search all databases from their inception to the present. Two review authors will independently extract data and perform risk of bias assessment. Primary outcomes will be the difference in mean depression scores on Hamilton Depression Rating Scale between the intervention and control groups and serious adverse events. Secondary outcomes will be suicide, suicide-attempts, suicidal ideation, quality of life and non-serious adverse events.

**Discussion:**

No former systematic review has systematically assessed the beneficial and harmful effects of duloxetine taking into account both the risks of random errors and the risks of systematic errors. Our review will help clinicians weigh the benefits of prescribing duloxetine against its adverse effects and make informed decisions.

**Systematic review registration:**

PROSPERO 2016 CRD42016053931

**Supplementary Information:**

The online version contains supplementary material available at 10.1186/s13643-021-01722-5.

## Background

### Depression

According to the World Health Organization (WHO), 264 million people suffer from depression around the globe [1] and major depressive disorder has been estimated to be the third leading cause of years lived with disability in both sexes [2]. Major depressive disorder is, depending on the diagnostic system, characterised by the occurrence of depressed mood, loss of interest or pleasure, reduced energy or fatigue accompanied by other symptoms such as suicidal thoughts, sleep disturbances, psychomotor agitation or retardation and difficulty concentrating [3, 4]. With a 12-month prevalence of around 5.5% in high-income countries [5], major depressive disorder is a large economic burden due to decreased work productivity [6] and it significantly impairs quality of life [7, 8].

### Antidepressants

Different classes of antidepressants are available for treatment of patients with major depressive disorder ranging from older antidepressants like mono-amine oxidase inhibitors (MAOI) and tri-cyclic antidepressants (TCA) to newer groups of drugs like selective serotonin reuptake inhibitors (SSRI) and serotonin-norepinephrine reuptake inhibitors (SNRI) as summarised in Table S1 (Additional file 1).

A report from the National Health and Nutrition Examination survey in the USA found an increase in the use of antidepressants from 7.7% in 1999–2002 to 12.7% in 2011–2014, wherein a quarter of those who took antidepressants had been using them for more than 10 years [9]. Whilst SSRIs remain the most commonly prescribed antidepressants, there has been a consistent increase in the prescription of other antidepressants such as duloxetine [10, 11].

### Duloxetine

Duloxetine, a SNRI, is approved for the treatment of major depressive disorder in the USA and Europe [12, 13]. It is, additionally, approved for a number of other conditions such as generalized anxiety, diabetic neuropathic pain, and fibromyalgia and is among the top 50 most prescribed drugs in the USA with the number of yearly prescriptions exceeding 16,000,000 [14]. In vivo and in vitro studies indicate that duloxetine inhibits the presynaptic neuronal reuptake of the neurotransmitters serotonin and norepinephrine, leading to their greater availability at the neuronal junctions and potentiating their action in the central nervous system [15, 16]. Serotonin and norepinephrine have been suggested to be involved in the pathogenesis of major depressive disorder [17], and theoretically the antidepressant effects of duloxetine have been speculated to be mediated through antagonising the depletion of these two neurotransmitters in the brain [18]. However, the potential role of these and other neurotransmitters in the pathophysiology and treatment of major depressive disorder is unclear [19, 20].

Duloxetine has an average half-life of around 12 h and is metabolised mainly in the liver [21]. Duloxetine is administered orally at a starting dose for the treatment of major depression of 60 mg/day, potentially increased to a maximum dose of 120 mg/day [13]. The most commonly reported adverse effects are nausea, dry mouth, decreased appetite, excessive sweating and drowsiness, whereas the most serious adverse effects include hepatic failure, orthostatic hypotension leading to syncope and falls, suicidal ideation, serotonin syndrome and increased risk of bleeding [22].

### Beneficial effects of duloxetine

Several previous reviews have shown that antidepressants seem to decrease depressive symptoms with a statistically significant effect [23, 24]. However, the effect is small and of uncertain clinical importance to patients [25]. A recent network meta-analysis including 23 trials on duloxetine reported for duloxetine versus placebo a standardised mean difference (SMD) of − 0.37 on depression scales. This was much lower than the empirically derived threshold of 0.875 SMD suggested by Moncrieff and Kirsch, corresponding to ‘minimal improvement’ on the Clinical Global Impressions-Improvement scale as well as lower than the less stringent criteria of 0.5 SMD, suggested by the National Institute of Clinical Excellence (NICE) in England [25–27]. More recently, Hengartner and Ploderl, reviewing both within patient and between patient anchor-based approaches, suggested that minimal important difference on 17-item Hamilton Depression Rating Scale (HDRS-17) is likely to be to be in the range of 3–5 points [28]. Whilst the ‘minimum clinically important difference’ on depression scales remains an area of debate with no consensus so far, the small statistically significant improvement in depressive symptoms with duloxetine must be weighed against the questionable clinical significance of the intervention whilst also taking harmful effects and costs into consideration.

Moreover, in many systematic reviews and meta-analyses including randomised placebo-controlled trials of duloxetine, remission and response defined as dichotomous outcomes were used as primary outcome measures and duloxetine was found to be superior compared with placebo (Table 1). However, dichotomisation of continuous scales to calculate response and remission has been criticised and might over-estimate the beneficial effects [25]. A decrease of only one point on the depression symptom severity scales can change categorisation of a trial participant from ‘non-remitter/non-responder’ to ‘remitter/responder’. It is therefore important to synthesise the evidence using the depression symptom severity scales without dichotomising the scores to assess the benefits associated with the use of antidepressants.

**Table 1:** Overview of previous reviews summarising benefits and/or harms of duloxetine versus placebo in participants with MDD

First author (year of publication)	Title	Design	Published protocol	Sources of information	No of trials	No of participants	Assessment of bias in included trials	Conclusions: remission/response duloxetine versus placebo	Conclusions: adverse effects duloxetine versus placebo
Krause et al. (2019) [29]	Efficacy and tolerability of pharmacological and non-pharmacological interventions in older patients with major depressive disorder: a systematic review, pairwise and network meta-analysis	Systematic review, pairwise and network meta-analysis	Yes	MEDLINE, Embase, PsycINFO, Cochrane Library, ClinicalTrials.gov, WHO registry, reference searches	4	1347	Yes	Superior: response defined as ≥ 50% reduction on HAM-D, MADRS, BDI or any other validated depression scale, score (1, 2) on CGI-improvement scale. Remission defined as ≤ 7 on HAM-D, ≤ 10 on MADRS, score (1,2) on CGI- severity scale, other criteria as defined in the primary trials.	Inferior: nausea, sedation, dizziness, diarrhea, hyperhidrosis, anticholinergic side effects, higher number of drop-outs due to adverse events.
Sobieraj et al. (2019) [30]	Adverse effects of pharmacologic treatments of major depression in older adults	Systematic review and meta-analysis	Yes	MEDLINE, Embase, Cochrane Central, PsycINFO, ClinicalTrials.gov, International Controlled Trials Registry	3	977	Yes	Not assessed	Superior with exception of falls. Higher number of drop-outs due to adverse events.
Cipriani et al. (2018) [24]	Comparative efficacy and acceptability of 21 antidepressant drugs for the acute treatment of adults with major depressive disorder: a systematic review and network meta-analysis	Systematic review and network meta-analysis	Yes	Cochrane Central Register of Controlled Trials, CINAHL, Embase, LILACS database, MEDLINE, MEDLINE In-Process, PsycINFO, AMED, UK National Research Register, ClinicalTrials.gov, websites of regulatory agencies, International Trial Registers	23	6733	Yes	Superior: response defined as ≥ 50% reduction of the total score on a standardised observer-rating scale for depression. Remission defined as ≤ 7 or 8 on HAM-D, ≤ 10 or 11 on MADRS or remission on any other standardised rating scale for depression.	Inferior: higher number of drop-outs due to adverse events.
Tham et al. (2016) [31]	Efficacy and tolerability of antidepressants in people aged 65 years or older with major depressive disorder—a systematic review and a meta-analysis	Systematic review and meta-analysis	Yes	PubMed, Embase, Cochrane Library, CINAL, PsycINFO, Scopus	3	977	Yes	Superior: response defined as ≥ 50% post-treatment reduction of scores on HAM-D-17, -21, -24 or MADRS-interview based. Remission was defined depending upon the depression scale used.	Inferior: dry mouth, constipation, diarrhea, dizziness.
Sharma et al. (2016) [32]	Suicidality and aggression during antidepressant treatment: systematic review and meta-analyses based on clinical study reports	Systematic review and meta-analysis	No	European and UK drug regulators, Eli Lilly’s website	12	Not clear	No (tools not sufficient)	Not assessed	No evidence of increased risk in adults (questionable due to quality of available data).
Thorlund et al. (2015) [33]	Comparative efficacy and safety of selective serotonin reuptake inhibitors and serotonin-norepinephrine reuptake inhibitors in older adults: a network meta-analysis	Network meta-analysis	No	MEDLINE, Embase, Cochrane Central Register of Controlled Trials, PsycINFO, Web of Science, ClinicalTrials.gov, conference proceedings of major psychiatric conferences for the past 2 years	1	311	No	Superior: partial response defined as 50% reduction on HAM-D or MADRS.	Inferior: dizziness.
Casale et al. (2012) [34]	Duloxetine in the treatment of elderly people with major depressive disorder	Systematic review	No	MEDLINE, Embase, PsycLIT	4	1132	No	Superior: reduction in scores on depression scales used in primary trials such as HAM-D-17 and HAM-D-24.	Slightly inferior: dry mouth, diarrhea, nausea, fatigue, insomnia, decreased appetite and libido.
Schueler et al. (2011) [35]	A systematic review of duloxetine and venlafaxine in major depression, including unpublished data	Meta-analysis	Yes	MEDLINE, Embase, PsycINFO, Psyndex, Cochrane Central Register of Controlled Trials, CDSR, DARE, Cochrane HTA, ClinicalTrials.gov, reference searches, unpublished data (Eli Lilly and Company)	12	3069	Yes	Superior: response and remission as defined in the primary trials.	Inferior: higher rate of discontinuation due to adverse events.
Nelson JC. (2010) [36]	Anxiety does not predict response to duloxetine in major depression: results of a pooled analysis of individual patient data from 11 placebo-controlled trials	Pooled analysis of individual patient data	No	Eli Lilly and Company sponsored trials	11	2841	No	Superior: response defined as ≥ 50% improvement on HAM-D. Remission defined as endpoint HAM-D score ≤ 7.	Not assessed
Mancini et al. (2010) [37]	Use of duloxetine in patients with an anxiety disorder or with comorbid anxiety and major depressive disorder: a review of the literature	Systematic review	No	MEDLINE, Embase	16	Not stated	No	Superior: HAM-D-17 total scores at end point, change in HAM-D.	Inferior: nausea, headache, dizziness, fatigue, suicidal thoughts, overdose.
Gartlehner et al. (2009) [38]	The general and comparative efficacy and safety of duloxetine in major depressive disorder: a systematic review and meta-analysis	Systematic review and meta-analysis	No	MEDLINE, Embase, PsychLIT, Cochrane Library, International Pharmaceutical Abstracts	11	Not stated	Yes	Superior: remission defined as endpoint score of ≤ 7 on HAMD-17), could not perform meta-analysis due to insufficient data.	Lack of definite evidence but increased risk for adverse effects.
Mukai et al. (2009) [39]	Treatment of depression in the elderly: a review of the recent literature on the efficacy of single- versus dual-action antidepressants	Systematic review	No	MEDLINE, PsycINFO, PubMed	1	311	No	Superior: change in HAM-D scores, GDS scores, cognitive score.	No difference in number of drop- outs.
Frampton et al. (2007) [40]	Duloxetine: a review of its use in the treatment of major depressive disorder	Systematic review	No	MEDLINE, Embase, AdisBase	8	1881	No	Superior: response defined as 50% reduction from baseline in HAM-D-17 scores at last observation. Remission defined as endpoint HAM-D score ≤ 7.	Inferior: nausea, dry mouth, constipation, insomnia, dizziness, fatigue, somnolence, decreased appetite, sexual dysfunction.
Mallinckrodt et al. (2006) [41]	Duloxetine for the treatment of major depressive disorder: a closer look at efficacy and safety data across the approved dose range	Pooled analyses	No	Eli Lilly and Company sponsored trials	4	868	No	Superior: mean change in HAM-D-17 total scores. Response defined as 50% reduction in HAM-D-17 total scores from baseline. Remission defined as HAM-D score ≤ 7.	Inferior: higher rate of discontinuation due to adverse events.
Acharya et al. (2006) [42]	Duloxetine: meta-analyses of suicidal behaviors and ideation in clinical trials for major depressive disorder	Meta-analysis	No	Eli Lilly and Company, Shionogi Company Ltd	12	2996	No	Not assessed	No evidence of increased risk.
Vis et al. (2005) [43]	Duloxetine and venlafaxine-XR in the treatment of major depressive disorder: a meta-analysis of randomized clinical trials	Meta-analysis	No	Cochrane, Embase, MEDLINE	6	1481	No	Superior: response defined as improvement of ≥ 50% from baseline on HAM-D or MADRS. Remission defined as HAM-D score ≤ 7 or MADRS ≤ 10.	Inferior: higher number of drop- outs due to adverse effects.

*BDI* Beck’s Depression Inventory, *CGI* clinical global impression, *HAM*-*D* Hamilton Depression Rating Scale, *GDS* Global Depression Scale, *MADRS* Montgomery Åsberg Depression Rating Scale, *MDD* major depressive disorder

### Harmful effects of duloxetine

In most reviews on duloxetine, adverse effects have not been sufficiently assessed. Instead, proxy measures like tolerability, acceptability and drop-outs due to adverse events have been used to assess safety profile of antidepressants compared with placebo [24, 35]. In other reviews, non-serious adverse events such as anticholinergic adverse effects, dizziness, nausea, sedation and hyperhidrosis have been frequently reported and discussed [29]. However, there is little to no information on more serious adverse events such as suicides or suicide attempts [29, 33, 34].

With regards to serious adverse events, some systematic reviews and meta-analyses report no increased risk of suicide or suicidal tendency with the use of antidepressants including duloxetine versus placebo in adult populations [37, 42], whilst others have observed an age-dependant increase in risk of suicidality [32, 44]. It is important to consider that these analyses suffered from incomplete reporting of adverse events in the included trials and limitations such as a lack of a pre-registered protocol [32, 44], no access to case-report forms [32] which are more likely to record adverse events in particular suicidal events as highlighted in other reviews [45] and low statistical power [42]. Moreover, some of the reviews on duloxetine were at risk of for-profit bias as the authors were employed at or the research was funded by the pharmaceutical industry [37, 42]. In one systematic analysis, Khan et al. examined safety data submitted to the U.S. Food and Drug Administration (FDA) during the period 1991–2013 for the approval of fourteen investigational antidepressants including duloxetine and reported a decline in suicide rates in the antidepressant groups of the clinical trials [46]. However, their analytical approach relied on patient-exposure years (PEY), which was deemed inappropriate by Hengartner and Ploderl [37]. They stressed that the risk of suicidal events is highest during the first few weeks of antidepressant use and this violates the *constant-hazard* requirement for a PEY analysis. Hengartner and Ploderl argued that this analytical approach can obscure the increased suicide risk associated with initiation of antidepressant use. They therefore reanalysed the data used by Khan et al. and found three times higher odds of suicide with the use of antidepressants [47]. The potential increase in suicide risk presented by Hengartner and Ploderl highlights the importance and need of evaluating adverse effects using appropriate methods.

A retrospective analysis of clinical study reports from 268 trials of drugs assessed by The German Institute for Quality and Efficiency in Health Care (IQWiG) between 2006 and Feb 2011 found that registry reports and publications were inferior to clinical study reports with regards to the outcome reporting, particularly of adverse events [48]. Another cross-sectional study of clinical trial registration summaries and their associated publications observed ambiguities and discrepancies in reporting of serious adverse events in journal articles and trial registration summaries [49]. A study comparing clinical study reports from nine randomised placebo-controlled trials of duloxetine with publicly available documents such as journal articles and results posted on trial registries found not only publication bias in favour of significant findings on efficacy analysis but also that information on serious adverse events was missing from journal articles and registry reports [50]. In addition, treatment emergent adverse effects were only reported in journal articles if the incidence was higher than a certain percentage, whereas information on discontinuation related adverse events was unavailable or vaguely reported if at all [50]. Another issue observed was that the coding of suicidality events from investigator reported adverse events resulted in inaccurate reporting of this information in clinical study reports as compared to the patient data [51]. Taken together, the evidence points to a need to extend the assessment of adverse events beyond published literature to get a more accurate assessment of benefits and harms associated with the use of antidepressants.

### Evidence assessments of duloxetine for major depressive disorder

We searched PubMed and Google Scholar for existing evidence on duloxetine using the search terms ‘duloxetine’, ‘major depression’ and ‘systematic reviews’. We identified a total of 16 meta-analyses, overviews or systematic reviews including randomised clinical trials on duloxetine versus placebo as summarised in Table 1 [36, 39–41, 43]. We identified three reviews and meta-analyses that summarised the evidence on both benefits and harms of duloxetine and which also assessed the risk of bias in the included trials. Only one of these reviews met all the criteria outlined in the Preferred Reporting Items for Systematic Reviews and Meta-Analyses (PRISMA) checklist [52]. However, this review was of low generalisability as it focussed only on elderly participants and only included trials published in English [31]. Furthermore, the review included only three duloxetine trials and no duloxetine-related serious adverse events were reported or discussed in this review. Of the other two reviews, one focussed on older adults only [29], and one included only four duloxetine trials, did not publish a protocol and limited electronic searches to English language [29, 38]. None of the reviews evaluated duloxetine versus ‘active placebo’, i.e. an active substance with no anti-depressant effect, e.g. an antihistamine that mimics the adverse effects of duloxetine such as dizziness, dry mouth and nausea [20, 22].

We also searched for ongoing systematic reviews comparing duloxetine versus ‘active’ placebo, placebo or no intervention for the treatment of major depression in the international prospective register of systematic reviews PROSPERO. We only found one protocol for a systematic review comparing duloxetine versus placebo; it plans to include trials where duloxetine was used for a wide range of indications apart from major depressive disorder such as generalized anxiety disorder, fibromyalgia and diabetic peripheral neuropathic pain [53]. Other identified ongoing systematic reviews on duloxetine will only include head-to-head comparisons with other antidepressants [54–56] or involve indications other than major depression [57]. We identified no systematic reviews assessing the benefits and harms of duloxetine compared with ‘active’ placebo.

Thus, no former or presently planned review has systematically reviewed the beneficial and harmful effects of duloxetine taking into account both the risk of random errors and the risk of systematic errors in all randomised clinical trials on major depressive disorder [58]. Hence, we planned this systematic review to assess the beneficial and harmful effects of duloxetine versus ‘active’ placebo, placebo or no intervention in the treatment of major depressive disorder. This review will also contribute data to a larger project assessing the beneficial and harmful effects of all anti-depressants in patients with major depressive disorder [59].

## Objectives

The objectives of this systematic review will be to assess the beneficial and harmful effects of duloxetine versus ‘active’ placebo, placebo or no intervention in adult participants with major depressive disorder.

## Methods

The protocol meets the reporting standards outlined in the Preferred Reporting Items for Systematic Review and Meta-Analysis Protocols (PRISMA-P) checklist (Additional file 2). The protocol was originally registered on PROSPERO in 2016, https://www.crd.york.ac.uk/prospero/display_record.php?RecordID=53931; however, because of non-availability of funds, the review process never started. The current protocol represents an updated version of the original protocol following the project revival in January 2020. We guarantee that data extraction has not started at the time of protocol submission to Systematic Reviews.

### Eligibility criteria

#### Trials

All randomised clinical trials comparing duloxetine with ‘active’ placebo, placebo or no intervention irrespective of publication type, publication status, publication year and language will be included. Quasi-randomised trials, e.g. trials using date of admission for allocating the participants, cluster randomised trials and observational studies will be excluded.

#### Participants

Adults as defined by the trialists with a primary diagnosis of major depressive disorder. The diagnosis of major depressive disorder must be based on one of the standardised criteria, from either International Classification of Diseases (ICD) 9, ICD 10 [4], ICD 11 [60], Diagnostic and Statistical Manual of mental disorders (DSM) III [61], DSM III-R [62], DSM IV, DSM IV-TR [63], DSM V [3] or Feighner criteria [64]. Trials exclusively including participants with a somatic disease and comorbid major depressive disorder and trials on major depressive disorder during or after pregnancy will be excluded as depression during or after pregnancy traditionally is investigated in separate trials; depression during or after pregnancy is theoretically influenced by hormonal changes and physical and psychological stress that may not be comparable to non-pregnant populations [65]. If only a subset of participants from a study is eligible, we will only include those that fulfil inclusion criteria provided data can be obtained for that specific group. We chose to include trials on adults only to avoid heterogeneity resulting from age of participants. Moreover, we focussed on major depressive disorders considering that it is a prevalent psychiatric disorder and a common indication for prescription of duloxetine [12].

#### Intervention

Duloxetine at any dose or duration.

#### Control

‘Active’ placebo, i.e. any active substance employed to mimic the adverse effects of taking duloxetine such as nausea, dry mouth, and dizziness.

Placebo, i.e. any ‘placebo’ substance containing no active substance.

No intervention, i.e. any control intervention with no treatment elements, e.g. ‘waiting list’. Our primary comparison of interest will be duloxetine versus ‘active placebo’. Secondarily, we will compare duloxetine versus placebo and no intervention, individually. We chose these comparisons as they represent real-life scenarios, e.g. placebo effect or effect of waiting for the treatment.

#### Co-interventions

Trials comparing duloxetine versus ‘active’ placebo, placebo or no intervention as add-on therapy to any other kind of intervention (e.g. treatment as usual or psychotherapy) will be included, but only if this co-intervention is described and delivered similarly in the intervention groups.

### Outcomes

#### Primary outcomes

- The difference between the mean values from the two intervention groups using the 17-item or the 21-item Hamilton Depression Rating Scale (HDRS) [66]. Where the 21-item scale is used, we will only include the result of the score based on the 17-item version.
- The proportion of participants with one or more serious adverse events. We will use the International Conference on Harmonization of technical requirements for registration of pharmaceuticals for human use—Good Clinical Practice (ICH-GCP) definition of a serious adverse event, which is any untoward medical occurrence that resulted in death, was life-threatening, required hospitalisation or prolonging of existing hospitalisation and resulted in persistent or significant disability or jeopardised the participant [67]. If the trialists do not use the ICH-GCP definition, we will include the data if the trialists use the term ‘serious adverse event’. If the trialists do not use the ICH-GCP definition nor use the term serious adverse event, then we will also include the data if the event clearly fulfils the ICH-GCP definition for a serious adverse event.

#### Secondary outcomes

- The proportion of participants with either a suicide or a suicide attempt (as defined by the trialists).
- Quality of life (assessed with any valid continuous quality of life scale such as quality of life in depression scale, EQ-5D or any other scale used by the trialists).
- Suicide ideation (assessed using, e.g. Columbia-Suicide Severity Rating Scale).

#### Exploratory outcomes

- The SDM [66] between the two intervention groups including trials that use any form of HDRS, Montgomery-Asberg Depression Rating Scale (MADRS) [68] or Beck’s Depression Inventory (BDI) [69]. If the trialists report other scales in addition to HDRS, we will use HDRS-17 in this meta-analysis. If HDRS-17 is not reported, we will use HDRS-21 followed by HDRS-6. Similarly, if the trials report both MADRS and BDI, we will use MADRS in the meta-analysis. We will back-calculate mean difference on HDRS from the SDM.
- The proportion of participants achieving response. We have defined response as a 50% reduction (from baseline) on either HDRS, MADRS or any other scale as used by trialists, in the stated order of preference.
- The proportion of participants achieving remission. We have, pragmatically, defined remission as a HDRS less than 8, MADRS less than 10 and BDI less than 10 points, in the stated order of preference.
- The proportion of participants with one or more adverse events not considered serious.
- The serious adverse events individually as stated by the trialists.
- The adverse events not considered serious individually as stated by the trialists.

We chose HDRS as the primary outcome in spite of its psychometric limitations as HDRS-17 is a commonly used assessment scale and recommended by international guidelines [70, 71]. Moreover, the minimal clinically important difference has been identified for HDRS-17 [26, 27].

Moreover, we do not intend to use SMD as the primary outcome as the underlying assumption as described in Cochrane Handbook for Systematic Reviews of Interventions is that ‘*the differences in SDs among studies reflect differences in measurement scales and not real differences in variability among study populations. If in two trials the true effect* (*as measured by the difference in means*) *is identical*, *but the SDs are different*, *then the SMDs will be different*. *This may be problematic in some circumstances where real differences in variability between the participants in different studies are expected*.’ [72]. We might observe variability in patients’ responses in these trials owing to the differences in inclusion criteria. For example, participants identified using different diagnostic criteria such as ICD 9 or DSM III that do not use operationalised criteria might differ from participants in other studies. Similarly, participants might differ in severity of depression at the time of inclusion, presence or absence of psychiatric co-morbidities or might come from different settings such as inpatient or outpatient departments.

#### Assessment time points

We will assess all outcomes at the end of treatment (our assessment time point of primary interest) as well as at maximum follow-up.

### Search methods

We will search the Cochrane Central Register of Controlled Trials (CENTRAL), MEDLINE, EMBASE, PsycInfo, Science Citation Index Expanded, Social Sciences Citation Index (SSCI), Conference Proceedings Citation Index—Science (CPCI-S) and Conference Proceedings Citation Index—Social Science & Humanities (CPCI-SSH) (Additional file 3). We will also search Chinese databases (CNKI, Wanfang, VIP, Sinomed) and Google Scholar. We will search all databases from their inception to present. We will check relevant publications, e.g. included trials and systematic reviews, for relevant trials. To identify unpublished trials, we will search trials registers of pharmaceutical companies, the WHO trial registry, clinicaltrials.gov, including the websites of the FDA and the European Medicines Agency (EMA). Furthermore, we will request clinical study reports from FDA, EMA and national medicines agencies. We will contact trial authors to seek required information.

### Screening of trials

Two of the review authors (FS and MB) will independently select relevant trials, based on criteria described in the above section. If a trial only has been identified by one of the two, it will be discussed whether the trial should be included. If the two review authors disagree, a third review author (JCJ) will decide if the trial should be included. All excluded trials assessed in full text will be entered on a list, stating the reason for exclusion.

### Data extraction

Data will be extracted by two reviewers independently. The following data will be extracted from the included trials:

1. Trial: publication status, date of publication, year of study conduction/randomisation, duration of trial, trial design, for-profit funding of trial, NCT/EudraCT number.

2. Participants: mean age, sex distribution, number randomised to each comparison group, number analysed, number lost to follow-up, drug or alcohol dependence, chronically depressed or treatment resistant depression (any definition used by the trialists), baseline depression scores, comorbid psychiatric diagnoses, borderline personality disorder, inclusion and exclusion criteria.

3. Intervention: length of intervention period and follow-up period, dose of duloxetine, dosing schedule, co-interventions such as psychotherapy or electroconvulsive therapy, whether the experimental intervention is an add-on therapy on other antidepressants, placebo washout period, choice of control (‘active’ placebo, placebo or no intervention).

4. Outcomes: primary and secondary outcomes (e.g. HDRS scores, BDI scores, number of suicides), type of outcome reported (e.g. change in scores, post-intervention scores), mean, standard deviation (SD) and number analysed for all continuous outcomes, number of events and number analysed for dichotomous outcomes, method of data collection for adverse effects, i.e. active monitoring or spontaneous report monitoring.

5. Others: author’s affiliations, an evaluation of the bias risk and choice of method (see below).

### Risk of systematic error (bias)

Two review authors will assess risk of bias in the included trials independent of each other using Cochrane’s risk of bias tool version 2 (RoB 2) [73]. The risk of bias assessment will be made for each outcome as well as overall risk of bias for the trial. We will evaluate the methodology to identify bias resulting from the randomisation process, deviation from the intended interventions, missing outcome data, measurement of the outcome as well as the selective reporting of results. We will classify the trials according to the components below as summarised in the RoB 2 guidance document (Table 2) [74].

**Table 2:** RoB 2 guidelines on risk of bias assessment

Bias arising from the randomisation process	
**Low risk of bias**	(i.) The allocation sequence was adequately concealed
	**AND**
	(ii.1) any baseline differences observed between intervention groups appear to be compatible with chance
	**OR**
	(ii.2) there is no information about baseline imbalances
	**AND**
	(iii.1) the allocation sequence was random
	**OR**
	(iii.2) there is no information about whether the allocation sequence was random
**Some concerns**	(i.1) The allocation sequence was adequately concealed
	**AND**
	(i.2.1) the allocation sequence was not random
	**OR**
	(i.2.2) baseline differences between intervention groups suggest a problem with the randomisation process
	**OR**
	(ii.1) there is no information about concealment of the allocation
	**AND**
	(ii.2) any baseline differences observed between intervention groups appear to be compatible with chance
	**OR**
	(iii) there is no information to answer any of the signalling questions
**High risk of bias**	(i.) The allocation sequence was not adequately concealed
	**OR**
	(ii.1) there is no information about concealment of the allocation sequence
	**AND**
	(ii.2) baseline differences between intervention groups suggest a problem with the randomisation process
Bias due to deviation from intended interventions	
**Low risk of bias**	(i.1) Participants, carers and people delivering the interventions were unaware of intervention groups during the trial
	**OR**
	(i.2.1) participants, carers or people delivering the interventions were aware of intervention groups
	**AND**
	(i.2.2) [if applicable] the important non-protocol interventions were balanced across intervention groups
	**AND** (ii) [if applicable] failures in implementing the intervention could not have affected the outcome
	**AND**
	(iii) [if applicable] study participants adhered to the assigned intervention regimen.
**Some concerns**	(i.1.1) Participants, carers and people delivering the interventions were unaware of intervention groups during the trial
	**AND**
	(i.1.2.1) [if applicable] failures in implementing the intervention could have affected the outcome
	**OR**
	(i.1.2.2) [if applicable] study participants did not adhere to the assigned intervention regimen
	**OR**
	(i.2.1) participants, carers or people delivering the interventions were aware of intervention groups and (i.2.2) [if applicable] the important non-protocol interventions were balanced across intervention groups
	**AND**
	(i.2.3.1) [if applicable] failures in implementing the intervention could have affected the outcome
	**OR**
	(i.2.3.2) [if applicable] study participants did not adhere to the assigned intervention regimen
	**OR**
	(i.3.1) participants, carers or people delivering the interventions were aware of intervention groups
	**AND**
	(i.3.2) [if applicable] the important non-protocol interventions were not balanced across intervention groups
	**AND**
	(ii) an appropriate analysis was used to estimate the effect of adhering to intervention.
**High risk of bias**	**(**i.1.1) Participants, carers and people delivering the interventions were unaware of intervention groups during the trial
	**AND**
	(i.1.2.1) [if applicable] failures in implementing the intervention could have affected the outcome
	**OR**
	(i.1.2.2) [if applicable] study participants did not adhere to the assigned intervention regimen
	**OR**
	(i.2.1) participants, carers or people delivering the interventions were aware of intervention groups
	**AND**
	(i.2.2) [if applicable] the important non-protocol interventions were balanced across intervention groups
	**AND**
	(i.2.3.1) [if applicable] failures in implementing the intervention could have affected the outcome
	**OR**
	(i.2.3.2) [if applicable] study participants did not adhere to the assigned intervention regimen
	**OR**
	(i.3.1) participants, carers or people delivering the interventions were aware of intervention groups
	**AND**
	(i.3.2) [if applicable] the important non-protocol interventions were not balanced across intervention groups
	**AND**
	(ii) an appropriate analysis was not used to estimate the effect of adhering to intervention
Bias due to missing outcome data	
**Low risk of bias**	(i.) Outcome data were available for all, or nearly all, randomised participants
	**OR**
	(ii.) there is evidence that the result was not biased by missing outcome data
	**OR**
	(iii) missingness in the outcome could not depend on its true value.
**Some concerns**	(i.) Outcome data were not available for all, or nearly all, randomized participants
	**AND**
	(ii.) there is not evidence that the result was not biased by missing outcome data
	**AND**
	(iii.) missingness in the outcome could depend on its true value
	**AND**
	(iv) it is not likely that missingness in the outcome depended on its true value.
**High risk of bias**	(i.) Outcome data were not available for all, or nearly all, randomized participants
	**AND**
	(ii.) there is not evidence that the result was not biased by missing outcome data
	**AND**
	missingness in the outcome could depend on its true value
	**AND**
	(iv) it is likely that missingness in the outcome depended on its true value
Bias in measurement of outcomes	
**Low risk of bias**	(i.) The method of measuring the outcome was not inappropriate
	**AND**
	(ii.) the measurement or ascertainment of the outcome did not differ between intervention groups
	**AND**
	(iii.1) the outcome assessors were unaware of the intervention received by study participants
	**OR**
	(iii.2) the assessment of the outcome could not have been influenced by knowledge of the intervention received.
**Some concerns**	(i.1) The method of measuring the outcome was not inappropriate
	**AND**
	(i.2) the measurement or ascertainment of the outcome did not differ between intervention groups
	**AND**
	(i.3) the assessment of the outcome could have been influenced by knowledge of the intervention received
	**AND**
	(i.4) it is unlikely that assessment of the outcome was influenced by knowledge of intervention received
	**OR**
	(ii.1) the method of measuring the outcome was not inappropriate
	**AND**
	(ii.2) there is no information on whether the measurement or ascertainment of the outcome could have differed between intervention groups
	**AND**
	(ii.3.1) the outcome assessors were unaware of the intervention received by study participants
	**OR**
	(ii.3.2) the assessment of the outcome could not have been influenced by knowledge of the intervention received.
**High risk of bias**	(i.) The method of measuring the outcome was inappropriate
	**OR**
	(ii.) the measurement or ascertainment of the outcome could have differed between intervention groups
	**OR**
	(iii) it is likely that assessment of the outcome was influenced by knowledge of the intervention received
Bias arising from selective reporting of results	
**Low risk of bias**	(i.) The data were analysed in accordance with a pre-specified plan that was finalised before unblinded outcome data were available for analysis
	**AND**
	(ii) the result being assessed is unlikely to have been selected, on the basis of the results, from multiple eligible outcome measurements (e.g. scales, definitions, time points) within the outcome domain
	**AND**
	(iii) reported outcome data are unlikely to have been selected, on the basis of the results, from multiple eligible analyses of the data
**Some concerns**	(i.1) The data were not analysed in accordance with a pre-specified plan that was finalised before unblinded outcome data were available for analysis
	**AND**
	(i.2) the result being assessed is unlikely to have been selected, on the basis of t he results, from multiple eligible outcome measurements (e.g. scales, definitions, time points) within the outcome domain
	**AND**
	(i.3) the result being assessed is unlikely to have been selected, on the basis of the results, from multiple eligible analyses of the data
	**OR**
	(ii) there is no information on whether the result being assessed is likely to have been selected, on the basis of the results, from multiple eligible outcome measurements (e.g. scales, definitions, time points) within the outcome domain and from multiple eligible analyses of the data.
**High risk of bias**	(i.) The result being assessed is likely to have been selected, on the basis of the results, from multiple eligible outcome measurements (e.g. scales, definitions, time points) within the outcome domain
	**OR**
	(ii) the result being assessed is likely to have been selected, on the basis of the results, from multiple eligible analyses of the data

### Overall assessment of risk of bias

*Low risk of bias*: The study is judged to be at low risk of bias for all domains for this result. *Some concerns*: The study is judged to be at some concerns in at least one domain for this result. *High risk of bias*: The study is judged to be at high risk of bias in at least one domain for this result OR the study is judged to have some concerns for multiple domains in a way that substantially lowers confidence in the result.

For our purposes, we will combine *some concerns* and *high risk of bias* judgements so that in our overall assessment of risk of bias, we will classify trials either to be at overall *low risk of bias* or at overall *high risk of bias*.

### Assessment of publication bias and for-profit bias

On all outcomes, we will create and inspect a funnel plot to assess possible small-study biases if ten or more trials are included, unless the trials are of similar size. For dichotomous outcomes, we will test asymmetry with the Harbord test if τ^2^ is less than 0.1 and with the Rücker test if τ^2^ is more than 0.1. For continuous outcomes, we will use the regression asymmetry test [75] and the adjusted rank correlation [76].

We will account for for-profit interests under publication bias in the GRADE assessment. Trials initiated, conducted or funded by pharmaceutical industry as well as the trials with any of the authors affiliated with the industry or where authors received grants from industry (self-reported in the article) will be considered at risk of for-profit interests [77]. We will downgrade for for-profit influence if the subgroup analysis according to risk of for-profit interests (see below) shows a difference between the intervention groups.

### Differences between the protocol and the review

The review will be conducted in accordance with this protocol. Deviations from the protocol, if any, will be reported in the systematic review under the section ‘Differences between the protocol and the review’.

### Statistical methods

Data will be meta-analysed using statistical software STATA 16.1 (StataCorp 2019. Stata Statistical Software: Release 16. College Station, TX: StataCorp LLC). We will undertake meta-analysis according to the recommendations stated in the Cochrane Handbook for Systematic Reviews of Interventions and the eight-step assessment suggested by Jakobsen et al. [58]. When analysing continuous outcomes, we will calculate mean differences (MDs) with 95% confidence intervals (CIs). We will use the Sidik-Jonkman model for random-effects meta-analysis [78]. We will also use the SMD with a 95% CI to analyse the results when different scales have been used. We will pool trials reporting change in scores and post intervention scores for mean difference; however, they will not be pooled for SMD [72]. We will also calculate trial sequential analysis-adjusted CIs (see below). When analysing dichotomous outcomes, we will calculate risk ratios (RRs) with 95% CI as well as the trial sequential analysis-adjusted CIs (see below). For rare outcomes such as adverse effects, we will use binomial regression analysis [79].

Intervention effects will be assessed by both random-effects model meta-analyses and fixed-effect model meta-analyses and we will use the more conservative point estimate of the two [72]. The more conservative point estimate is the estimate with the highest *P* value. We plan to assess a total of five primary and secondary outcome therefore we will consider *P* ≤ 0.016 as statistically significant [58]. We will investigate possible heterogeneity through subgroup analyses. We will use the eight-step procedure to assess if the thresholds for significance are crossed [58]. Our primary conclusion will be based on trials at overall low risk of bias. Where multiple trial arms are reported in a single trial, we will include only the relevant arms. For trials with multiple intervention arms, we will correspondingly divide the control group. If quantitative synthesis is not appropriate due to considerable heterogeneity or a small number of included trials, we will report the results in a descriptive way.

Although there is no current consensus on the issue, the National Institute for Clinical Excellence (NICE) of the National Health Service in England has formerly defined a threshold for clinical significance for major depressive disorder as an effect size of 0.50 SMD or a drug-placebo difference of three points on the 17-item HDRS [27]. Others have suggested and used the following ‘rule of thumb’: 0.2 SMD represents a small effect, 0.5 SMD a moderate effect and 0.8 SMD a large effect [72, 80]. We have chosen, as NICE has formerly recommended and other reviewers have chosen [27, 81, 82], a drug-placebo difference of three points on the 17-item HDRS (for our primary outcome) or an effect size of 0.50 SMD (for our exploratory outcome) as the threshold for clinical significance. This is in line with findings from a recent review, suggesting that the most likely minimal important difference on the HDRS-17 is between 3 and 5 points [28].

To control the risk of type I and type II errors, we will use Trial Sequential Analyses. We will perform trial sequential analyses on all the outcomes [83–85], in order to calculate the diversity-adjusted required information size (that is, the number of participants needed in a meta-analysis to detect or reject a certain intervention effect) and the cumulative Z-curve’s breach of relevant trial sequential monitoring boundaries. A more detailed description of trial sequential analysis can be found at http://www.ctu.dk/tsa [84]. For continuous outcomes, trial sequential analysis will use the empirical SD, a mean difference of three points on the Hamilton Depression Rating Scale (17 or 21 item) and the observed SD/2 when other depression scales or quality of life scales are used, an alpha of 1.7%, a beta of 10% and adjustment for the observed diversity. For dichotomous outcomes, trial sequential analysis will use the proportion of participants with an outcome in the control group, a relative risk reduction of 25%, an alpha of 1.7% for primary outcomes, a beta of 10% and adjustment for the observed diversity of the trials in the meta-analysis.

### Missing outcomes

We will use intention-to-treat data if reported by the trialists [86]. If intention-to-treat data are not reported, we will use the data as reported by the trialists. We will, as the first option, contact all trial authors to obtain any relevant missing data (i.e. for data extraction and for assessment of risk of bias, as specified above).

Dichotomous outcomes: we will not impute missing values for any outcomes in our primary analysis. In our sensitivity analyses (see paragraph below), we will impute data.

Continuous outcomes: we will primarily analyse scores assessed at single time points (end scores). If only changes from baseline scores are reported, we will analyse the results together with end scores [72]. If SDs are not reported, we will calculate the SDs using trial data, if possible. We will not use intention-to-treat data if the original report did not contain such data. We will not impute missing values for any outcomes in our primary analysis. In our sensitivity analysis (see paragraph below) for continuous outcomes, we will impute data.

### Sensitivity analyses

To assess the potential impact of the missing data for dichotomous outcomes, we will perform the following two sensitivity analyses on both the primary and the secondary dichotomous outcomes. We will present results of both scenarios in our review.

- ‘Best-worst-case’ scenario: we will assume that all participants lost to follow-up in the antidepressant group had a beneficial outcome, i.e. survived, had no serious adverse events, had no suicides or suicide attempts and had no non-serious adverse events, and that all those participants lost to follow-up in the control group had a harmful outcome, i.e. did not survive, had a serious adverse event, died by suicide or had a suicide attempt and had a non-serious adverse event.
- ‘Worst-best-case’ scenario: we will assume that all participants lost to follow-up in the antidepressant group had a harmful outcome, i.e. did not survive, had a serious adverse event, died by suicide or had a suicide attempt and had a non-serious adverse event, and that all those participants lost to follow-up in the control group had a beneficial outcome, i.e. survived, had no serious adverse events, had no suicides or suicide attempts and had no non-serious adverse events. When analysing continuous outcomes like depressive symptoms and quality of life, a ‘beneficial outcome’ will be reduction in depression scores and increase in quality of life scale and will be calculated as group (intervention or control) mean plus two SDs (we will secondly use one SD in another sensitivity analysis) of the group mean. Similarly, ‘harmful outcome’ will be increase in depression scores and decrease in quality of life scale and will be calculated as the group mean minus two SDs (we will secondly use one SD in another sensitivity analysis) of the group mean [68]. This data imputation with 2 SDs will provide a possible range of influence that missing data might have on the results [87]. To assess the potential impact of missing data for continuous outcomes, we will perform the following sensitivity analysis:
- Where SDs are missing and it is not possible to calculate them, we will impute SDs from trials with similar populations and low risk of bias. If we find no such trials, we will impute SDs from trials with a similar population. As the final option, we will impute SDs from all trials.
- We will perform sensitivity analysis to assess the effect of using ICH-GCP definition of serious adverse events.

We will present results of these scenarios in our review. Other post hoc sensitivity analyses might be warranted if unexpected clinical or statistical heterogeneity is identified during the analysis of the review results.

### Assessment of heterogeneity

We will primarily investigate forest plots to visually assess any sign of heterogeneity. We will secondly assess the presence of statistical heterogeneity by chi^2^ test (threshold *P* < 0.10) and measure the quantities of heterogeneity by the *I*^2^ statistic. We will investigate possible heterogeneity through subgroup analyses. According to the Cochrane Handbook, *I*^2^ statistic above 50% will be regarded as substantial heterogeneity and we may ultimately decide that a meta-analysis should be avoided [72].

### Subgroup analyses

We have planned the following subgroup analyses on all the outcomes:

1. Whether the intervention effects from trials at overall low risk of bias (or lower risk of bias) differ from the trials at overall high risk of bias as it can potentially over-estimate beneficial effects or bias the estimates for harmful effects towards the null.
2. Whether the intervention effects from the trials using ‘active’ placebo, placebo, or no intervention differ.
3. Whether the results from trials using a placebo washout period before inclusion differ from the remaining trials.
4. Whether the intervention effects of duloxetine differ in trials at low risks of *for*-*profit* interests compared to trials at high risks of *for*-*profit* interests [77].
5. Whether the intervention effects from trials assessing the effects of duloxetine in elderly depressive participants (defined by the trialists but often adults ≥ 65 years) differ from the remaining trials.
6. Whether the intervention effects from trials with participants with a baseline HDRS score of 23 or above differ from the remaining trials as intervention effect might vary depending upon baseline scores.
7. Whether the intervention effects from trials assessing the effects of duloxetine in chronically depressive patients or treatment resistant depression differ from the remaining trials.
8. Whether the intervention effect differ by duration of treatment, i.e. the trials with duration of treatment below 6 weeks, between 6 and 12 weeks and above 12 weeks. Since the intervention duration could be an important determinant of intervention effect. If a trial reports multiple time-points within these groups, we will use the longest time period.
9. Duloxetine below or equal to median dose compared to above median dose.
10. Whether the intervention effect differ depending upon the scale used in the trial, i.e. HDRS, MADRS or BDI.

### GRADE

We will assess the certainty of evidence of all outcomes using GRADE (Grading of Recommendations Assessment, Development and Evaluation) tool. Cochrane Handbook for Systematic Reviews of Interventions (Chapter 8: Section 8.5 and Chapter 12) will be followed for GRADE evaluation using the GRADEpro software [72]. We will use the five GRADE domains (bias risk of the trials, consistency of effect, imprecision, indirectness and publication bias) to assess the quality of a body of evidence. Imprecision will be assessed using trial sequential analysis [58]. We will downgrade imprecision in GRADE by two levels if the accrued number of participants is below 50% of the diversity-adjusted required information size (DARIS), and one level if between 50% and 100% of DARIS. We will not downgrade if the cumulative Z-curve crosses the monitoring boundaries for benefit, harm or futility, or DARIS is reached [88]. The findings for primary outcomes will be presented in a summary of findings table where each GRADE domain will be presented for trials contributing data to the meta-analyses for the prespecified outcomes [58, 89]. We will justify all decisions when downgrading the certainty of evidence using footnotes, and we will add comments to aid the reader’s understanding of the review where necessary.

## Discussion

One major strength of this protocol is that we aim to compare benefits and harmful effects of duloxetine versus ‘active’ placebo, placebo or no intervention in adult participants with major depressive disorder. This is a strength as few earlier reviews have addressed both harms and benefits, and serious adverse events have not been sufficiently analysed in these reviews as demonstrated in our ‘Background’ section. Considering that the use of antidepressants is associated with several short-term and long-term adverse effects, it is critical to review available evidence and to establish if harms outweigh the benefits associated with the use of antidepressants.

Another strength of this protocol is its methodological approach. We will follow the recommendations outlined in the Cochrane Handbook for Systematic Reviews of Interventions [72]. We will use the eight-step assessment suggested by Jakobsen et al. [58], trial sequential analysis [84] and the GRADE assessment of the certainty of evidence [89] to assess clinical significance of our findings as well as to address the risks of random and systematic errors and to establish the quality of evidence.

The primary limitation of our systematic review is the potential for heterogeneity as a result of methodological variability in the included trials. To minimise this limitation, we will carefully look for signs of heterogeneity and ultimately decide if data ought to be pooled and meta-analysed, and we have planned several subgroup analyses.

Another limitation is the large number of comparisons which increases the risk of type 1 error. We have adjusted our thresholds for significance according to the number of primary and secondary outcomes, but we have not adjusted our thresholds for significance according to the number of subgroup analyses.

Another potential limitation is the insufficiency of adverse effect reporting in the published literature [90, 91]. To address that, we will request clinical study reports from FDA, EMA, other national medicines agencies as well as from the pharmaceutical companies as they are likely to contain more information on adverse effects compared to trial registries and published articles.

In a similar vein, we have decided only to include randomised clinical trials and exclude quasi-randomised studies and observational studies. Through these decisions, we run the risks of overlooking late as well as rare adverse effects. If we find benefits of duloxetine that is not overpowered by adverse events in the randomised clinical trials, someone needs to the assess the risks of adverse events according to quasi-randomised trials and observational studies [92].

## Supplementary Information

**Additional file 1:.** Table 1. Types of antidepressants.

**Additional file 2..** PRISMA-P check list.

**Additional file 3..** Search strategies for duloxetine for major depressive disorder.

## Data Availability

Data sharing is not applicable to this protocol article. We will publish relevant data in the supplementary material of the systematic review.

## Abbreviations

BDI: Beck’s Depression Inventory
DSM: Diagnostic and Statistical Manual of Mental Disorders
EMA: European Medicines Agency
FDA: Food and Drug Administration
HDRS: Hamilton Depression Rating Scale
ICD: International Classification of Diseases
MADRS: Montgomery-Asberg Depression Rating Scale
MAOI: Mono-amine oxidase inhibitors
NICE: National Institute of Clinical Excellence
PEY: Patient-exposure years
PRISMA: Preferred Reporting Items for Systematic Reviews and Meta-Analyses
SD: Standard deviation
SMD: Standardised mean difference
SNRI: Serotonin-norepinephrine reuptake inhibitors
SSRI: Selective serotonin reuptake inhibitors
TCA: Tri-cyclic antidepressants
WHO: World Health Organization
