# Supplementary material for: Duloxetine versus ‘active’ placebo, placebo or no intervention for major depressive disorder; a protocol for a systematic review of randomised clinical trials with meta-analysis and trial sequential analysis
Source: Syst Rev. 2021 Jun 9;10:171. doi: 10.1186/s13643-021-01722-5 (PMC8191126; doi:10.1186/s13643-021-01722-5)
Supplement: Supplementary file 3 — Additional file 3.. Search strategies for duloxetine for major depressive disorder. [file 13643_2021_1722_MOESM3_ESM.pdf]

Search strategies for duloxetine for major depressive disorder

Cochrane Central Register of Controlled Trials (CENTRAL) in the Cochrane Library

#1 MeSH descriptor: [Duloxetine Hydrochloride] explode all trees
#2 (duloxetin* or cymbalta* or Irenka*)
#3 #1 or #2
#4 MeSH descriptor: [Depressive Disorder, Major] explode all trees
#5 MeSH descriptor: [Depressive Disorder] this term only
#6 MeSH descriptor: [Seasonal Affective Disorder] explode all trees
#7 MeSH descriptor: [Dysthymic Disorder] explode all trees
#8 MeSH descriptor: [Depression] explode all trees
#9 MeSH descriptor: [Affective Symptoms] this term only
#10 (MDD or depress* or ((affective or adjustment or dysthym* or mood) and (disorder* or disease* or symptom*)))
#11 #4 or #5 or #6 or #7 or #8 or #9 or #10
#12 #3 and #11

MEDLINE Ovid (1946 to the date of the search)

1. exp Duloxetine Hydrochloride/
2. (duloxetin* or cymbalta* or Irenka*).mp. [mp=title, abstract, original title, name of substance word, subject heading word, keyword heading word, protocol supplementary concept word, rare disease supplementary concept word, unique identifier]
3. 1 or 2
4. exp Depressive Disorder, Major/
5. Depressive Disorder/
6. exp Seasonal Affective Disorder/
7. exp Dysthymic Disorder/
8. exp Depression/
9. Affective Symptoms/
10. (MDD or depress* or ((affective or adjustment or dysthym* or mood) and (disorder* or disease* or symptom*))).mp. [mp=title, abstract, original title, name of substance word, subject heading word, keyword heading word, protocol supplementary concept word, rare disease supplementary concept word, unique identifier]
11. 4 or 5 or 6 or 7 or 8 or 9 or 10
12. 3 and 11
13. (randomized controlled trial or controlled clinical trial).pt. or clinical trials as topic.sh. or trial.ti.
14. (random* or blind* or placebo* or meta-analys*).mp. [mp=title, abstract, original title, name of substance word, subject heading word, floating sub-heading word, keyword heading word, organism supplementary concept word, protocol supplementary concept word, rare disease supplementary concept word, unique identifier, synonyms]
15. 12 and (13 or 14)

Embase Ovid (1974 to the date of the search)

1. exp duloxetine/
2. (duloxetin* or cymbalta* or Irenka*).mp. [mp=title, abstract, heading word, drug trade name, original title, device manufacturer, drug manufacturer, device trade name, keyword, floating subheading]
3. 1 or 2
4. exp major depression/
5. depression/
6. exp seasonal affective disorder/
7. exp dysthymia/
8. emotional disorder/
9. (MDD or depress* or ((affective or adjustment or dysthym* or mood) and (disorder* or disease* or symptom*))).mp. [mp=title, abstract, heading word, drug trade name, original title, device manufacturer, drug manufacturer, device trade name, keyword, floating subheading]

10. 4 or 5 or 6 or 7 or 8 or 9
11. 3 and 10
12. Randomized controlled trial/ or Controlled clinical study/ or trial.ti.
13. (random* or blind* or placebo* or meta-analys*).mp. [mp=title, abstract, heading word, drug trade name, original title, device manufacturer, drug manufacturer, device trade name, keyword, floating subheading word, candidate term word]
14. 11 and (12 or 13)

PsycINFO (1806 to the date of the search)
1. (duloxetin* or cymbalta* or Irenka*).mp. [mp=title, abstract, heading word, table of contents, key concepts, original title, tests & measures]
2. exp Major Depression/
3. exp Seasonal Affective Disorder/
4. exp Dysthymic Disorder/
5. exp "DEPRESSION (EMOTION)"/
6. exp Affective Disorders/
7. (MDD or depress* or ((affective or adjustment or dysthym* or mood) and (disorder* or disease* or symptom*))).mp. [mp=title, abstract, heading word, table of contents, key concepts, original title, tests & measures]
8. 2 or 3 or 4 or 5 or 6 or 7
9. 1 and 8
10. (random* or blind* or placebo* or meta-analys*).mp. [mp=title, abstract, heading word, table of contents, key concepts, original title, tests & measures]
11. 9 and 10

LILACS (Bireme; 1982 to the date of the search)
(duloxetin$ or cymbalta$ or Irenka$) [Words] AND (MDD or depress$ or ((affective or adjustment or dysthym$ or mood) and (disorder$ or disease$ or symptom$))) [Words]

Science Citation Index Expanded (Web of Science; 1900 to the date of the search) and Conference Proceedings Citation Index – Science (Web of Science; 1990 to the date of the search)
#1 TS=(duloxetin* or cymbalta* or Irenka*)
#2 TS=(MDD or depress* or ((affective or adjustment or dysthym* or mood) and (disorder* or disease* or symptom*)))
#3 #1 AND #2
#4 TI=(random* or blind* or placebo* or meta-analys* or trial*) OR TS=(random* or blind* or placebo* or meta-analys*)
#5 #3 AND #4
